# Supplementary material for: Ranking versus rating in peer review of research grant applications
Source: PLoS One. 2023 Oct 5;18(10):e0292306. doi: 10.1371/journal.pone.0292306 (PMC10553257; doi:10.1371/journal.pone.0292306)
Supplement: Attachment — Submitted filename: Response to reviews 2022-11-09.docx [file pone.0292306.s001.docx]

**Ranking versus Rating in Peer Review of Research Grant Applications**

Response to Reviews

| **Reviewer Comment** | **Authors’ Response** |
| --- | --- |
| 1. Perhaps the most convincing result in the paper is that ranking increases the reliability of the review compared to raw scores (Table 3). However, the mechanism articulated by the authors is a bit vague to me. The authors claimed that the benefit of ranking stems from removing the requirement for a stable internalized standard, which is difficult for inexperienced reviewers compared to experienced reviewers. This seems an overly complicated explanation. A more straightforward explanation would be that ranking controls for leniency (1) variation among evaluators, so the evaluation is more robust. Either way, I recommend a more thorough discussion of potential mechanisms and perhaps lay out future tests that can distinguish between these hypotheses. | The theory underlying decision-making processes is based on decades of research in cognitive science which has defined two types of decision-making: absolute (rating) and comparative (ranking). As these theories have been applied to successfully explain decision-making in a wide variety disciplines and applications, it is reasonable to assume that it is relevant in the context of peer review. We have added another possible mechanism to the discussion of our findings—specifically that ranking forces ties to be broken, when applications have the same absolute score. This would effectively increase the between application variance relative to the within rater variance thereby increasing reliability. |
| 2. Compared to the results about reliability, the results about reducing bias (table 5) are much less convincing. The main reason is that the quality proxy for proposals is H-index. While this might be partially justified in the first stage evaluation (since the first stage appears to be evaluating people, not the grant proposal), it is important to acknowledge that H-index can be a signal of prestige instead of researcher quality, so the results in table 5 might as well reflect that ranking and rating weighted different kinds of bias differently; | We agree with the points raised by the reviewer and acknowledged the limitations of using the H-index as a measure of quality in the second last paragraph of the discussion. |
| 3. Given that the authors found relatively convincing evidence of ranking reducing reliability versus reducing bias, it might be beneficial to clearly contrast these two concepts in the introduction, as many readers can lump the two concepts together and believe they are concordant measures of peer-review quality. Sometimes, larger variance in evaluation can be good for better evaluation outcomes since pooling different opinions can result in the ‘wisdom of the crowd (2,3)’; | Thank-you for this suggestion. We have now added description of bias versus reliability to the introduction. |
| Comments about statistics:  1. I have a minor concern about ICC measures for reliability (I might be wrong since I am not an expert on this measure). If I remember correctly, the ICC requires some normality assumption (4). This is likely true for raw rating score but might not be valid for percentile ranking, as one would expect the percentile ranking would follow a uniform distribution between 0-1. Or maybe it will be approximately normal after taking the mean? | Just like a correlation, or a proportion, or any other bounded measure, the sampling variation can be non-symmetric, especially if the sample size is small  -- and indeed we use transformations (such as logits, or Fisher's z statistic for the regular correlation, to try to have the sampling  distributions be more Gaussian.  But in this case, with very large numbers of degrees of freedom for both components of the icc, the 'central limit theorem is operating, and the sampling variation is very close to Gaussian. And we see this is borne out in the bootstrap (re-sampling) procedure. |
| 2. If I understand correctly, the regressions in Tables 4 and 5 are also used to search for factors that are significantly associated with variance (another way of measuring reliability) and outcome in different stages and schemes. If this is the case, the authors are effectively conducting multiple testing. In this case, a good practice is to perform multiple testing corrections, even for multiple regression (5). At the very least, authors can display the p-values with higher precision so that interested readers can gauge the results when needed. Along the same lines, if figure 1 shows the only significant interaction terms, then the (adjusted) p-value of the interaction term should be demonstrated, as figure 1 is what remains among the several interaction terms tested as described in the main text. Besides, to show the non-linear effect in figure 1, confidence intervals should be plotted; | With respect to the results from the multiple regression models in Table 4 and 5, we had planned these magnitude estimations ahead of time with just this set of predictors.  We show the CIs to show how much each factor contributes beyond what the others already contribute. Finally, we have added an extra decimal place to the p-values, so that those who see this as opportunistic searching can adjust their interpretations accordingly |
| Comment about presentation:  1. Finally, the organization of the paper should be substantially improved for readability. For example, a schematic figure showing the reviewing process of the CIHR program would be helpful. Furthermore, the formula for multiple regressions conducted should be explicitly shown instead of relying on a verbal description. In addition, the results would be much easier to read if divided into different sections (with titles) according to the conclusions. | Thank-you for your suggestions. We have added subtitles to demarcate sections in the results. The formula for the multiple regression models is included in the footnote of Table 4 and Table 5. |
