# Supplementary material for: Ranking versus rating in peer review of research grant applications
Source: PLoS One. 2023 Oct 5;18(10):e0292306. doi: 10.1371/journal.pone.0292306 (PMC10553257; doi:10.1371/journal.pone.0292306)
Supplement: Attachment — Submitted filename: Response to Reviewers-PLOS-2023-05-04.docx [file pone.0292306.s002.docx]

**Reviewer #1: The authors have addressed all my comments. I have no further questions. The only suggestion is that the formula presentation could possibly be improved via LaTex. But I assume this can be done in the final proofreading process.**

*The equations in the table footnotes have been re-formatted in LaTex*

**Reviewer #2: The authors suggest that the peer review process used in determining the funding priority of submitted proposals has been plagued by reports of poor reliability and that one potential solution may lie in examining other schemas of evaluation, including a ranking process, which may be more reliable and may be less prone to bias. To examine this, the authors compare the reliability associated with application rating with those of application ranking in 3,156 applications that have been submitted to the Canadian Institutes of Health Research funding agency. In general, the authors found that ranking was more reliable than rating and “less susceptible to the characteristics of the review panel, such as level of expertise and experience, for both reliability and potential sources of bias.” This natural experiment is important and the authors should be lauded for taking this analysis on, as access to these types of data are limited. In general, this is an important area, and improving upon current review processes is important not only to provide more consistent output but for the credibility of the scientific process itself.**

*Thank-you for your comments and support for this work.*

**First, the differences observed in the reliability measures for rating and ranking are an important finding. While it seems the rating and ranking are independent processes, they are also highly correlated. Is there an assumption here that reviewers use the same criteria for ranking and rating? If they are different, do the authors have any insight into the ways they diverge? It may be helpful to add a little language in the discussion addressing what the authors think might be the differences in the decision-making processes peer reviewers use for rating vs ranking?**

*The advantage of this natural experiment was that the criteria used to rate and rank applications were the same. While we have no information on the decision-making process of reviewers , we have hypothesized possible mechanisms that could be operating in the ranking process that would lead to improved reliability and reduction in bias to the discussion.*

**Also, the reliabilities for rating and ranking are somewhat similar for stage 1 and better than that of stage 2 (possibly because reviewers in stage 1 are mostly discriminating between good versus bad proposals?). For stage 2, reliability for both rating and ranking is worse, but the difference between them has grown, suggesting ranking is more clearly advantageous over rating for stage 2 (perhaps because judgements at this stage are more difficult; i.e. it is more difficult to discriminate between good and great applications via a rating mechanism?). Do the authors feel the utility of ranking over rating improves for proposals that are in the good/great zone? This is important as this may be a range where peer review is the least reliable or at least has the most difficulty in discriminating between proposals and may be most prone to subjectivity and bias.

Another area that might be undersold here a bit is the inherent power of ranking for tie-breaking. Could the authors elaborate on this in the analysis (e.g. how many rating ties are broken by the ranking process?).**

*This is an excellent point and we have added this information on tie breaking to the results reported relative to Table 3. As the reviewer suspects, tie breaking was a frequent occurrence. In phase 1, the average number of ties that needed to be broken by a reviewer were for 31.7% of their application reviews and only 16% of reviewers had no ties that needed to be broken. In phase 2,ties needed to eb broken in 28.3% of applications and by 75.4% of reviewers.*

**While a good portion of the analysis is spent on bias, it is not entirely clear that ranking is devoid of bias or if it is less susceptible to bias, why that is in the context of peer review? Maybe the authors might add a little language in the discussion to address this.**

*We agree. In our discussion we indicated that ranking may confer reductions in potential sources of bias, we clarified that this comment referred to our findings related to applicant gender and early career status where there was an appreciable difference in the performance of rating and ranking.*

**A minor concern, the authors state that “Ranking would also provide greater flexibility in recruiting reviewers with different levels of experience and expertise without compromising reliability.” But is the goal to reduce expertise levels of reviewers?**

*We realize this sentence is misleading and have revised it as follows..*

*“Ranking would also provide greater flexibility in recruiting reviewers from various disciplines with different levels of experience without compromising reliability”*

**Also, the authors mention the online discussion increased ranking variance “With respect to the review process, we found that on-line discussion of an application was associated with higher rater variance, which may be expected, as efforts would be selectively made to resolve differences in review opinion among reviewers with very divergent scores” Maybe the authors could provide more description about how proposals were selected for online discussion (I’m presuming there was a cut-off for discrepant scores?). If one looked at scores before and after discussion, would you would see a resolving of scores, i.e. scores coming together (reduced variance)?**

*In the Methods section, we have augmented the description of the peer review process to indicate how applicants were selected for on-line discussion. The reviewer has raised an important point about the potential consequences of discussion on rater variance. Unfortunately only the final rating and ranking were retained by the peer review system so this question cannot be addressed.*

**Reviewer #3: The authors present the work titled: "Ranking versus rating in peer review of research grant applications." Though the topic is of great relevance, it has several flaws that authors should address to enhance the quality of the manuscript.

First, the literature review omits several relevant aspects that the reader should know to understand the full scope and implications of the research. Note, however, that the literature on grant funding evaluations is enormous. A potential contribution in this regard relates to a comprehensive literature review. Unfortunately, the literature review provided by the authors is weak. The authors could have included several other factors to illustrate or explain their empirical strategy. For example, the Matthew effect (Bol et al, 2018), gender effects (Eloy et al, 2013), the use of multi-criteria approaches (Oztaysi et al, 2017), the effects per discipline (Jerrim & Vries, 2020); implementation considerations (Neta et al 2015), or previous recommendations for reliability and validity (Marsh et al 2008) are just a few points of an extensive list of missed points.**

*We thank the reviewer for identifying additional references that were not identified in our literature search, many of which were relevant to the research question addressed in this manuscript. The literature review outlined in the introduction was related to the specific question of reliability and bias in grant applications using two methods, rating and ranking. We cover both the theoretical literature on these forms of assessment, and the results of applications in other contexts as well as in grant review. A comprehensive literature review on all that is known about grant peer review was beyond the scope or intent of this manuscript. Two comprehensive reviews have already been published on this topic, both of which are cited in this manuscript (Guthrie, 2018; Shepherd, 2018).*

**Another concern is the statistical treatment. In particular, the authors have missed potential problems associated with endogeneity in the data. Endogeneity broadly refers to situations in which an explanatory variable (e.g., the H-Index of the applicant) correlates with the error term of the regression equation (tables 4 and 5). In this case, if authors do not control for how many co-authors an applicant has worked with in previous works, this might increase the error term of the regression equation because H-index correlates with the number of co-authors (Vinkler, 2023).**

*This is a very good point. We have redone the analysis to include the number of unique collaborators listed in the applicant’s publications to address this problem of unmeasured covariates in the error term. The correlation between the H index and number of collaborators is reasonably high (r=0.5). Both the H index and number of collaborators are significantly associated with rater variance and the score in the same direction. However, there is no impact of including number of collaborators on estimates of applicant, reviewer and review characteristics on variance and bias. The new results are presented in Table 4 and 5.*

**Due to the issues described above, the manuscript can not be accepted in its current form and the authors should address them before an additional consideration for the study.**


SUGGESTED REFERENCES
Bol, T., de Vaan, M., & van de Rijt, A. (2018). The Matthew effect in science funding. Proceedings of the National Academy of Sciences, 115(19), 4887-4890.

Eloy, J. A., Svider, P. F., Kovalerchik, O., Baredes, S., Kalyoussef, E., & Chandrasekhar, S. S. (2013). Gender differences in successful NIH grant funding in otolaryngology. Otolaryngology--Head and Neck Surgery, 149(1), 77-83.

Jerrim, J., & Vries, R. D. (2020). Are peer-reviews of grant proposals reliable? An analysis of Economic and Social Research Council (ESRC) funding applications. The Social Science Journal, 1-19.

Marsh, H. W., Jayasinghe, U. W., & Bond, N. W. (2008). Improving the peer-review process for grant applications: reliability, validity, bias, and generalizability. American psychologist, 63(3), 160.

Neta, G., Sanchez, M. A., Chambers, D. A., Phillips, S. M., Leyva, B., Cynkin, L., ... & Vinson, C. (2015). Implementation science in cancer prevention and control: a decade of grant funding by the National Cancer Institute and future directions. Implementation Science, 10, 1-10.

Oztaysi, B., Onar, S. C., Goztepe, K., & Kahraman, C. (2017). Evaluation of research proposals for grant funding using interval-valued intuitionistic fuzzy sets. Soft Computing, 21, 1203-1218.

Vinkler, P. (2023). Impact of the number and rank of coauthors on h-index and π-index. The part-impact method. Scientometrics, 1-21.
