# Supplementary material for: Ranking versus rating in peer review of research grant applications
Source: PLoS One. 2023 Oct 5;18(10):e0292306. doi: 10.1371/journal.pone.0292306 (PMC10553257; doi:10.1371/journal.pone.0292306)
Supplement: Attachment — Submitted filename: Editor response + review response.docx [file pone.0292306.s003.docx]

Dear Editor,

Thank you for providing us with the opportunity to respond to comments from a new reviewer of our manuscript. If I am to understand correctly, our responses to the previous sets of reviewers were acceptable but the most recent reviewer has raised new issues not previously identified by the prior sets of reviewers.

Unfortunately, the critique provided by this reviewer was based on a series of assumptions that are incorrect. They include the following:

1. *The reviewer has assumed that the unit of analysis is the reviewer (comment 1,2). This is incorrect.* The unit of analysis is **the application**. We measured the characteristics of the application and its score and percentile rank based on the mean of ratings and percentile ranks provided by all reviewers of the application.
2. *The reviewer has assumed that there is a range restriction in scores in phase 2, as was the case in the Erosheva paper that was cited by the reviewer (comment 3). This is incorrect.* The applications in phase 2 were scored again, using a different set of reviewers and criteria to assess the quality of the research program using the the full score range for their assessment. Our analysis is based on ALL applications, both those that were funded and those that were unsuccessful in both Phase 1 and Phase 2.
3. *The reviewer assumed that the data were ordinal, likely because of confusion about the unit of analysis (comment 5). This is incorrect.* The data are continuous as evidenced by the histogram and rug plot shown in response to this comment. Each application received a rating allowing one decimal place of a scale of 0-112 for phase 1 and 0-140 for phase 2 and a ranking from each of the 5 reviewers which was converted by CIHR to a percentile ranking to adjust for differences in the number of applications rated by each reviewer. The arithmetic mean of these values became the application score and percentile rank.
4. *The reviewer assumed that only bivariate analysis was conducted (comment 7).* *This is incorrect.*  In the analysis section we indicated that multivariate regression was used to estimate the associations between application, applicant and reviewer characteristics and each outcome, and provided the regression models in the footnotes of each table. By definition, multivariate models provide estimates of the independent association of a given variable, controlling for all other variables in the model.

Our possibly unclear description of the methodology may have led, in part, to this series of incorrect assumptions. Therefore, we have revised the methods section of the paper to improve clarity and avoid these misunderstandings by future readers.

Our detailed responses to each comment follow.

*The authors consider an important problem and attempt to study it using a large grant peer review data set from a reputable funding agency. The question of comparative reliability in ranking and rating is interesting. However, the actual quality assessment procedure that is being studied relative to standard rating is not ranking; it could be more accurately described as “tie-breaking score-based rank percentile” procedure. Therefore, the manuscript’s title and abstract claims are misleading.

Is the manuscript technically sound, and do the data support the conclusions?*

*There are substantial technical concerns with the manuscript that do not allow one to determine if the data support the conclusions about the use of ranking in grant peer review. The most important concerns include:

1. Although the title and abstract state that reliability of ratings and rankings are compared in the manuscript, that is not the case. Instead, the authors compare reliability of ratings and rank percentiles, obtained after reviewers were asked to break ties in their ratings. Rank percentiles are substantially different from rankings and have distinct properties. Many desirable properties of rankings, such as the direct comparison of proposals without reference to a scale, are lost when converting ranks to rank percentiles.*

We have clarified our description of the process of ranking in the methodology and provided the following example in an on-line appendix. We are unsure what the reviewer means by the comment “Many desirable properties of rankings, such as the direct comparison of proposals without reference to a scale, are lost when converting ranks to rank percentiles”. The grant peer reviewers never worked with percentile ranks, only with the rank order of applications. They were asked to order all applications from best to worst, breaking ties as appropriate. The ranking process had no scale. We provide examples to illustrate how the grant peer reviewers changed the rank order of the applications and how they broke ties.

It was only after the grant peer reviewer completed the ranking exercise that CIHR converted the ranks to percentile ranks. This conversion was necessary because reviewers of the same application may have reviewed a different number of applications. The mean of the percentile ranks provided by each reviewer of the application was the final application score.

Appendix A provides three de-identified examples from the data set used in the analysis.

**In Example 1,** Reviewer A reviewed 14 applications and scored them from 18 to 106 for the highest score application. The reviewer was presented with the initial ranking based on score. Two applications were tied with a score of 70. The reviewer broke this tie providing application #6 with an adjusted rank of 8 and application #7 with a rank of 9. Reviewer A also changed the rank order of application #13 and #14, from a rank of first to application #14 to second and application #13 was ranked first. CIHR converted the adjusted ranks to rank percentiles. If reviewer was the unit of analysis the mean rank percentile, by definition, would be 50% (as illustrated). However, in our study application was the unit of analysis. To obtain an overall score the application the mean of the percentile ranks of each reviewer was calculated. In example 1, for application #6, there were 5 reviewers with initial scores ranging from 70 to 96, and percentile ranks from 41.177 to 75.00 providing an overall mean percentile rank score of 54.549.

| **UNIT OF ANALYSIS IS THE REVIEWER** | | | | | | | | | | |
| --- | --- | --- | --- | --- | --- | --- | --- | --- | --- | --- |
| **Example Scores and percentile ranks for REVIEWER A and the 14 applications he was assigned to** | | | | | | | | | | |
| **Application number** | **Reviewer pin** | | **Reviewers initial score** | | **Reviewer INITIAL Rank** | | **Reviewer ADJUSTED rank** | | | **Reviewers percentile rank** |
| 1 | A | | 18 | | 13 | | 14 | | | 0.000 |
| 2 | A | | 48 | | 12 | | 13 | | | 7.692 |
| 3 | A | | 54 | | 11 | | 12 | | | 15.385 |
| 4 | A | | 60 | | 10 | | 11 | | | 23.077 |
| 5 | A | | 62 | | 9 | | 10 | | | 30.769 |
| 6 | A | | 70 | | 8 | | 8 | | | 46.154 |
| 7 | A | | 70 | | 8 | | 9 | | | 38.462 |
| 8 | A | | 74 | | 7 | | 7 | | | 53.846 |
| 9 | A | | 80 | | 6 | | 6 | | | 61.539 |
| 10 | A | | 84 | | 5 | | 5 | | | 69.231 |
| 11 | A | | 88 | | 4 | | 4 | | | 76.923 |
| 12 | A | | 92 | | 3 | | 3 | | | 84.615 |
| 13 | A | | 98 | | 2 | | 1 | | | 100.000 |
| 14 | A | | 106 | | 1 | | 2 | | | 92.308 |
|  |  | | **Mean percentile rank** | |  | |  | | | **50.000** |
| **UNIT OF ANALYSIS IS THE APPLICATION** | | | | | | | | |  |  |
| **Scores and percentile ranks for APPLICATION no 6 from the 5 REVIEWERS assigned to review it & Mean percentile rank** | | | | | | | | |  |  |
| **Application number** | | **Reviewer pin** | | **Reviewers initial score** | | **Reviewers percentile rank** | | **Mean percentile rank** |  |  |
| 6 | | a | | 70 | | 46.154 | | 54.549 |  |  |
| 6 | | b | | 80 | | 41.177 | | 54.549 |  |  |
| 6 | | c | | 84 | | 66.667 | | 54.549 |  |  |
| 6 | | d | | 84 | | 43.750 | | 54.549 |  |  |
| 6 | | e | | 96 | | 75.000 | | 54.549 |  |  |
|  | |  | | **Mean percentile rank** | | **54.549** | |  |  |  |

**In Example #2,** Reviewer Z reviewed 16 applications, scoring them from 64 to 106 with one tied score for application #15 and #16 of 76. The reviewer broke this tie ordering application #15 with a rank of 11 and #16 with a rank of 12. The lowest ranked application was #11 with an adjusted rank of 16 and a percentile rank of 0.00. Application #11 had 5 reviewers who scored the application between 48 and 84 with percentile ranks ranging from 0.00 to 62.5. The mean percentile rank score for this application was 28.56.

| **Example Scores and percentile ranks for REVIEWER Z and the 16 applications he was assigned to** | | | | | |
| --- | --- | --- | --- | --- | --- |
| **Application number** | **Reviewer pin** | **Reviewers initial score** | **Reviewer INITIAL Rank** | **Reviewer ADJUSTED rank** | **Reviewers percentile rank** |
| 11 | Z | 64 | 15 | 16 | 0.000 |
| 12 | Z | 68 | 14 | 15 | 6.667 |
| 13 | Z | 70 | 13 | 14 | 13.333 |
| 14 | Z | 72 | 12 | 13 | 20.000 |
| 15 | Z | 76 | 11 | 11 | 33.333 |
| 16 | Z | 76 | 11 | 12 | 26.667 |
| 17 | Z | 86 | 10 | 10 | 40.000 |
| 18 | Z | 88 | 9 | 9 | 46.667 |
| 19 | Z | 90 | 8 | 8 | 53.333 |
| 20 | Z | 92 | 7 | 7 | 60.000 |
| 21 | Z | 96 | 6 | 6 | 66.667 |
| 22 | Z | 98 | 5 | 5 | 73.333 |
| 23 | Z | 100 | 4 | 4 | 80.000 |
| 24 | Z | 102 | 3 | 3 | 86.667 |
| 25 | Z | 104 | 2 | 2 | 93.333 |
| 26 | Z | 106 | 1 | 1 | 100.000 |
|  |  | **Mean percentile rank** |  |  | **50.000** |

| **Scores and percentile ranks for APPLICATION no 11 from the 5 REVIEWERS assigned to review it & Mean percentile rank** | | | | |
| --- | --- | --- | --- | --- |
| **Application number** | **Reviewer pin** | **Reviewers initial score** | **Reviewers percentile rank** | **Mean percentile rank** |
| 11 | Z | 64 | 0.000 | 28.560 |
| 11 | P | 62 | 13.333 | 28.560 |
| 11 | Q | 84 | 62.500 | 28.560 |
| 11 | T | 70 | 31.250 | 28.560 |
| 11 | Y | 48 | 35.714 | 28.560 |
|  |  | **Mean percentile rank** | **28.559** |  |

**In Example #3,** Reviewer T reviewed 13 applications, scoring them from 78 to 112. The reviewer broke two ties involving application #36 and #37 as well as #38 and #39. In addition, the reviewer changed the rank order of the top three applications, moving the third ranked to the second. The top ranked application for Reviewer T (application #43) had 5 reviewers, four of whom ranked the application as first (rank percentile=100.00) in the pool of applications they reviewed, providing an overall mean percentile rank from the 5 reviewers of 87.50 for application #43.

| **Example Scores and percentile ranks for REVIEWER T and the 13 applications he was assigned to** | | | | | |
| --- | --- | --- | --- | --- | --- |
| **Application number** | **Reviewer pin** | **Reviewers initial score** | **Reviewer INITIAL Rank** | **Reviewer ADJUSTED rank** | **Reviewers percentile rank** |
| 31 | T | 78 | 11 | 13 | 0.000 |
| 32 | T | 84 | 10 | 12 | 8.333 |
| 33 | T | 88 | 9 | 11 | 16.667 |
| 34 | T | 96 | 8 | 10 | 25.000 |
| 35 | T | 98 | 7 | 9 | 33.333 |
| 36 | T | 100 | 6 | 7 | 66.667 |
| 37 | T | 100 | 6 | 8 | 58.333 |
| 38 | T | 102 | 5 | 5 | 50.000 |
| 39 | T | 102 | 5 | 6 | 41.667 |
| 40 | T | 104 | 4 | 4 | 75.000 |
| 41 | T | 106 | 3 | 2 | 91.667 |
| 42 | T | 108 | 2 | 3 | 83.333 |
| 43 | T | 112 | 1 | 1 | 100.000 |
|  |  | **Mean percentile rank** |  |  | **50.000** |

| **Scores and percentile ranks for APPLICATION no 43 from the 5 REVIEWERS assigned to review it & Mean percentile rank** | | | | |
| --- | --- | --- | --- | --- |
| **Application number** | **Reviewer pin** | **Reviewers initial score** | **Reviewers percentile rank** | **Mean percentile rank** |
| 43 | T | 112 | 100.000 | 97.500 |
| 43 | EE | 106 | 87.500 | 97.500 |
| 43 | Q | 100 | 100.000 | 97.500 |
| 43 | A | 112 | 100.000 | 97.500 |
| 43 | V | 104 | 100.000 | 97.500 |
|  |  | **Mean percentile rank** | **97.500** |  |

*2. Relatedly, some conclusions noted by the authors are the direct mathematical result of the construction of their “ranking” data. Such conclusions include: (1) the authors note on line 240 that the mean rank percentile is 50%, which is a consequence of their data conversion that would hold for any data source.*

The mean rank percentile is, by construction, is 50% when the unit of analysis is the **reviewer** as shown in our three examples Our study used the **application** as the unit of analysis. The final score for the application was the mean of the percentile ranks provided by all reviewers of the application. As illustrated in the three examples, the mean percentile rank of the application is, of course, not 50%.

We did not conduct any conversion of the data. The rating, ranking, adjusted ranking, and percentile ranking of each reviewer for an application, and the final mean ranking for each application is provided by CIHR. We have all ranks and percentile ranks for all reviewers for all applications for each stage of the competition. No grants were excluded from the analysis.

*(2) Similarly, the lack of statistical significance for coefficients in the rank percentile model is likely the result of the “zero-sum” construction of rank percentiles, and not necessarily a true lack of statistical significance.*

The zero-sum construction of rank percentiles would only apply if we were using reviewer as the unit of analysis (see above examples). We wish to point out that the zero-sum construction does not apply when using application as the unit of analysis where the outcome is the mean of all reviewers’ percentile ranks.

*3. Although authors note that “inclusion of only top scoring applications in phase 2 reduced the reliability of assessment”, they still make the conclusion on lines 356-360 that IRR was found to be smaller for the assessment of research project quality (phase 2) than that of researchers (phase 1). This comparison is flawed. That IRR decreased in phase 2 is primarily a mathematical result of removing the estimated “worst” proposals (see Erosheva, Martinkova, and Lee, 2021).*

We apologize for the lack of clarity in the presentation of results. We think the reviewer has assumed that the same phase 1 score was used to calculate the IRR for applications that were successful in reaching phase 2. This is not correct. In phase 2, a different set of reviewers was selected and they used a different set of criteria and scoring system to rate the quality of the research program, using the full range of possible scores. We have revised this paragraph describing the results to simply report that the ICC was lower for phase 2. In the discussion we have outlined likely reasons; 1) that the quality of candidates selected to submit their research program to phase 2 was more homogeneous, making it more difficult for reviewers to distinguish amongst higher quality proposals, and/or 2) that there is less agreement among reviewers about what constitutes a high quality research program than there is for evaluating the quality of the candidate (that was evaluated in phase 1).

*4. On lines 138-139, the authors note “it was assumed that the distribution of the quality of applications assigned to each reviewer would be equivalent.” What is precisely meant by the phrase “the distribution of the quality of applications…would be equivalent”? Whatever the formal description of this assumption might be, it appears to be quite stringent and unlikely to hold, especially if there was substantial variability in the number of proposals assessed by each reviewer. The authors report the mean numbers of 12.6 and 9.5 of proposals assessed by each reviewer in phases 1 and 2, respectively, but do not comment on the range. Given this implausible assumption, the authors should explore its influence on the results.*

In the “ideal” world, **all** reviewers would review **each** application submitted to a given program/committee, and the average rank for these applications would be assigned to the application. However this is a completely impractical. Nor is it possible to randomly allocate applications to reviewers whereby the random assignment would ensure that an equivalent number of poor, good and excellent applications would be reviewed by each reviewer. The priority in CIHR’s assignment of applications to reviewers was to optimize the match between reviewer expertise and the content of the application. *A priori,* neither CIHR or the chair of a committee would have knowledge of the quality of the application, therefore there was no possibility of systematic bias in assignment (for example where only the poor applications would go to one reviewer and the excellent applications to another). Therefore, it is assumed that, on balance, reviewers would obtain a mix of applications of varying quality. Any violation of this assumption would contribute to random errors in measurement and lead to an under-estimate of the reliability of ranking. We have noted this limitation in the discussion. The range in the number of applications assigned to a reviewer has been added to the description of Table 1 results.

*5. Comparing the reliability (variance) of ratings and rankings is ultimately challenging given the ordinal properties of rankings. The authors’ attempts to make this comparison, while commendable in goal, are not statistically appropriate.*

We include the histogram and rug plots of the log of the inter-rater variance for individual application scores and ranks for analyses reported in Table 3, to clarify that the data are normally distributed and continuous. Confusion about the nature of the data may have occurred if the reviewer assumed that the reviewer was the unit of analyses in the study. In our study, application is the unit of analysis. Each application has four to five reviewers, each of whom scored the application.

**Distribution of the Variance of Ranks between Raters of an Application**

**Distribution of the Variance of Scores between Raters of an Application**

*6. On numerous instances, we believe the authors use the phrase “percent rank” when referring to “rank percentile”. These are different concepts.*

This was our error, and we have corrected our terminology throughout the manuscript.

*7. Interpretation of regression coefficients should be done conditionally on all other variables being held constant.*

In the analysis section we indicated that multivariate regression was used to estimate the associations between application, applicant and reviewer characteristics and each outcome, and provided the regression models in the footnotes of each table. By definition, multivariate models provide estimates of the independent association of a given variable, controlling for all other variables in the model.

*Is the manuscript presented in an intelligible fashion and written in standard English?

Yes.*

References:

Erosheva, E. A., Martinková, P., & Lee, C. J. (2021). When zero may not be zero: A cautionary note on the use of inter‐rater reliability in evaluating grant peer review. Journal of the Royal Statistical Society: Series A (Statistics in Society)

Has the statistical analysis been performed appropriately and rigorously?

Please refer to the concerns raised in answering the previous question.

Have the authors made all data underlying the findings in their manuscript fully available?

The data will not be publicly available citing privacy concerns. We suggest the authors attempt to release a de-identified subset of the data publicly, as has been done with similar data from the Swiss NSF in Heyard et al. (2021), the National Institutes of Health in Erosheva et al. (2020), and the American Institute of Biological Sciences in Pearce and Erosheva (2022).

References:

Heyard, R., Ott, M., Salanti, G., & Egger, M. (2022). Rethinking the funding line at the Swiss national science foundation: Bayesian ranking and lottery. Statistics and Public Policy, 9(1), 110-121.

Erosheva, E. A., Grant, S., Chen, M. C., Lindner, M. D., Nakamura, R. K., & Lee, C. J. (2020). NIH peer review: Criterion scores completely account for racial disparities in overall impact scores. Science Advances, 6(23), eaaz4868.

Pearce, M., & Erosheva, E. A. (2022). A unified statistical learning model for rankings and scores with application to grant panel review. Journal of Machine Learning Research, 23(210), 1-33.
