# Supplementary material for: Ranking versus rating in peer review of research grant applications
Source: PLoS One. 2023 Oct 5;18(10):e0292306. doi: 10.1371/journal.pone.0292306 (PMC10553257; doi:10.1371/journal.pone.0292306)
Supplement: Attachment — Submitted filename: Response to Reviews-september-2023.docx [file pone.0292306.s004.docx]

**Dear author/s, thanks for submitting your work to PLoS ONE,
All major conceptual and methodological reviews of the article have been addressed. Still, please consider the following minor revisions:**

**- Add a space (•) before cited references. This applies to all the manuscript (e.g., from “investigators(4-8)” to “investigators•(4-8)”**

*We have corrected the spacing issue throughout the manuscript.*

- **Authors mention in section “Applicant characteristics” that “For each publication, we retrieved the citation reports and assigned the impact factor for each journal by linking the ISSN of the journal to the Journal Citation Record file. When there was no recorded ISSN, we used the full and abbreviated journal name to make the link.” It is not clear why the authors sourced the impact factor of journals if the computation of the H-Index do not require such a value (H-Index=for a set of articles N of an author and defining ci as the number of citations corresponding to an article i then ordering the set of articles in decreasing order according to the number of citations).**

*This was our oversight as we had another measure we were working on for other projects that required the journal impact factor. Thank-you so much for picking up this error. We have corrected it in our revised manuscript.*

**In addition, although at this point it might be out of the article scope as it is, would be to include an H-Index based indicator that controls for an authors number of collaborators which is the hm index (For a set of articles N with ci the number of citations for the article i and ai the number of corresponding authors, the cumulative sum of the inverse of the number of authors is proposed as the effective rank . Then, sorting the set of articles in decreasing order according to the number of citations:** [**https://doi.org/10.1016/j.joi.2008.05.001**](https://doi.org/10.1016/j.joi.2008.05.001)**). The advantage is to compute an H index based indicator that controls for the number of collaborators throughout a researcher's career.**

*Thank you for this suggestion for a minor revision. However, based on the comment from a previous peer review, we have decided to keep the number of collaborators as a separate variable that we adjust for in the models to transparently address the reviewer’s concerns about endogeneity.*

**Prior Reviewer: Another concern is the statistical treatment. In particular, the authors have missed potential problems associated with endogeneity in the data. Endogeneity broadly refers to situations in which an explanatory variable (e.g., the H-Index of the applicant) correlates with the error term of the regression equation (tables 4 and 5). In this case, if authors do not control for how many co-authors an applicant has worked with in previous works, this might increase the error term of the regression equation because H-index correlates with the number of co-authors (Vinkler, 2023).**

*Our response to the prior review: This is a very good point. We have redone the analysis to include the number of unique collaborators listed in the applicant’s publications to address this problem of unmeasured covariates in the error term. The correlation between the H index and number of collaborators is reasonably high (r=0.5). Both the H index and number of collaborators are significantly associated with rater variance and the score in the same direction. However, there is no impact of including number of collaborators on estimates of applicant, reviewer and review characteristics on variance and bias. The new results are presented in Table 4 and 5.*

**- PLoS ONE strongly advocates for a data availability and reproducible results as much as possible. Authors stated in “Data availability” item: “No - some restrictions will apply” considering the Canadian Institutes for Health Research (CIHR) policy. However, it might be available by request to the Vice-President of Research Programs-Operations at CIHR. Ultimately, I strongly suggest, if possible, to the authors to share in a public/institutional repository the code scripts produced for the analysis so other authors with the approval from the Canadian Institutes for Health Research (CIHR) could be able to reproduce/replicate/crowdsource the results of the study.**

We have noted that the SAS code used to conduct the analyses will be available at McGill -Dataverse curated by the McGill University Library (McGill University Dataverse (borealisdata.ca) within the next 2 weeks

Sincerely,

Julian D. Cortés
Associated editor

**Another concern is the statistical treatment. In particular, the authors have missed potential problems associated with endogeneity in the data. Endogeneity broadly refers to situations in which an explanatory variable (e.g., the H-Index of the applicant) correlates with the error term of the regression equation (tables 4 and 5). In this case, if authors do not control for how many co-authors an applicant has worked with in previous works, this might increase the error term of the regression equation because H-index correlates with the number of co-authors (Vinkler, 2023).**

*This is a very good point. We have redone the analysis to include the number of unique collaborators listed in the applicant’s publications to address this problem of unmeasured covariates in the error term. The correlation between the H index and number of collaborators is reasonably high (r=0.5). Both the H index and number of collaborators are significantly associated with rater variance and the score in the same direction. However, there is no impact of including number of collaborators on estimates of applicant, reviewer and review characteristics on variance and bias. The new results are presented in Table 4 and 5.*
